# Mental health status and substance abuse among students at ‘Ismail Qemali’ University of Vlora, Albania

**DOI:** 10.1192/j.eurpsy.2026.10813

**Published:** 2026-07-31

**Authors:** E. Shaska, E. Totraku, I. Kuka, E. Isuf, E. Roshi, F. Mulita

**Affiliations:** 1 “Ali Mihali” Psychiatric Hospital, Vlora; 2 University of Medicine, Tirana, Albania; 3 University of Patras, Patras, Greece

## Abstract

**Introduction:**

Many research publications have shown that smoking, alcohol, and drug use not only increase health risks in young people, for example, in the cardiovascular system, but also cause mental health issues like anxiety and stress. This study was conducted with students of the “Ismail Qemali” University of Vlora.

**Objectives:**

In this prospective study, we wanted to see the association between the usage of alcohol, smoking, and drugs and the stress and anxiety levels in everyday life. Additionally, we wanted to explore how the background influences this usage.

**Methods:**

We used a questionnaire that asked the following: gender, age, faculty, year of study, socio-economic status, father’s and mother’s level of education and employment’s status, if they smoke, drink alcohol, use cannabis, cocaine, heroin or amphetamines and how much and at the end how much stress and anxiety were felt in the last two weeks.

**Results:**

825 students were questioned. Of which 79% were female. Most people were living in urban areas, and specifically 72,40% in Vlore. Stress levels were average, with the 35,88% in the question ‘how often have you felt like you were unable to control the important things in your life?’ answered sometimes, the 30,54% answered often in the question ‘how often have you felt confident in your abilities to cope with personal problems?’, the 34,06% answered sometimes in the question ‘how often have you felt that things were going the way you wanted?’ and the 34,91% also answered sometimes in the question ‘how often have you felt that your difficulties have increased, so much so that you cannot cope with them?’.Anxiety levels were relatively low because the majority of people answered sometimes in the questions ‘have you felt nervous, anxious, or impatient?’ with percentage of 46,67%, have you felt like you couldn’t control your worries?’ with 39,51%, ‘have you felt excessively worried about things?’ with 38,79%, ‘have you had difficulty relaxing?’ with 40,12%. Moreover, in the question ‘have you felt so anxious that you couldn’t stand still?’ with 45,94% answering never, have you felt irritable or upset?’ with 41,33% answering sometimes, and ‘have you felt afraid because something terrible might happen?’ with 47,15% answering never.

**Conclusions:**

Results show that low levels of drug, alcohol and smoking use are associated with low levels of stress and anxiety, which points out the significance of healthy habits for mental health among students.

**Disclosure of Interest:**

None Declared

